# Stereotactic body radiotherapy with CyberKnife^®^ System for low- and intermediate-risk prostate cancer: clinical outcomes and toxicities of CyPro Trial

**DOI:** 10.3389/fonc.2023.1270498

**Published:** 2023-11-07

**Authors:** Valentina Borzillo, Esmeralda Scipilliti, Donato Pezzulla, Marcello Serra, Gianluca Ametrano, Giuseppe Quarto, Sisto Perdonà, Sabrina Rossetti, Sandro Pignata, Anna Crispo, Piergiacomo Di Gennaro, Valentina D’Alesio, Cecilia Arrichiello, Francesca Buonanno, Simona Mercogliano, Antonio Russo, Antonio Tufano, Rossella Di Franco, Paolo Muto

**Affiliations:** ^1^ Department of Radiation Oncology, Istituto Nazionale Tumori—IRCCS—Fondazione G. Pascale, Napoli, Italy; ^2^ Radiation Oncology Unit, Responsible Research Hospital, Campobasso, Italy; ^3^ Department of Uro-Gynecological, Istituto Nazionale Tumori—IRCCS—Fondazione G. Pascale, Napoli, Italy; ^4^ Departmental Unit of Clinical and Experimental Uro-Andrologic Oncology, Istituto Nazionale Tumori—IRCCS—Fondazione G. Pascale, Napoli, Italy; ^5^ Epidemiology and Biostatistics Unit, Istituto Nazionale Tumori—IRCCS—Fondazione G. Pascale, Napoli, Italy; ^6^ LB Business Services SRL, Rome, Italy; ^7^ Department of Diagnostic Imaging and Radiation Oncology, University “Federico II” of Naples, Napoli, Italy; ^8^ Department of Maternal-Child and Urological Sciences, Policlinico Umberto I Hospital, Sapienza University, Rome, Italy

**Keywords:** clinical outcome, toxicity, low-and intermediate-risk, radiotherapy, prostate cancer, stereotactic body radiation

## Abstract

**Simple summary:**

Stereotactic body radiotherapy (SBRT) of 35–36.25 Gy in five fractions with the CyberKnife System yields excellent control with low toxicity in low–intermediate-risk prostate cancer patients. We found no differences in biochemical control and overall survival in relation to dose. There were no significant differences in toxicity or quality of life between the two groups.

**Aims:**

Stereotactic body radiotherapy (SBRT) is an emerging therapeutic approach for low- and intermediate-risk prostate cancer. We present retrospective data on biochemical control, toxicity, and quality of life of CyPro Trial.

**Materials and methods:**

A total of 122 patients with low- and intermediate-risk prostate cancer were treated with the CyberKnife System at a dose of 35 Gy or 36.25 Gy in five fractions. Biochemical failure (BF)/biochemical disease-free survival (bDFS) was defined using the Phoenix method (nadir + 2 ng/ml). Acute/late rectal and urinary toxicities were assessed by the Radiation Therapy Oncology Group (RTOG) toxicity scale. Quality of life (QoL) was assessed by the European Organisation for Research and Treatment of Cancer (EORTC) QLQ C30 and PR25. International Erectile Function Index-5 (IIEF5) and International Prostate Symptom Score (IPSS) questionnaires were administered at baseline, every 3 months after treatment during the first years, and then at 24 months and 36 months.

**Results:**

The 1-, 2-, and 5-year DFS rates were 92.9%, 92.9%, and 92.3%, respectively, while the 1-, 2-, and 5-year bDFS rates were 100%, 100%, and 95.7%, respectively. With regard to risk groups or doses, no statistically significant differences were found in terms of DFS or bDFS. Grade 2 urinary toxicity was acute in 10% and delayed in 2% of patients. No Grade 3 acute and late urinary toxicity was observed. Grade 2 rectal toxicity was acute in 8% and late in 1% of patients. No Grade 3–4 acute and late rectal toxicity was observed. Grade 2 acute toxicity appeared higher in the high-dose group (20% in the 36.25-Gy group versus 3% in the 35-Gy group) but was not statistically significant.

**Conclusion:**

Our study confirms that SBRT of 35–36.25 Gy in five fractions with the CyberKnife System produces excellent control with low toxicity in patients with low–intermediate-risk prostate cancer. We found no dose-related differences in biochemical control and overall survival. Further confirmation of these results is awaited through the prospective phase of this study, which is still ongoing.

## 1 Introduction

A variety of treatment options for patients with localized prostate cancer (LPC) are available such as surgery, external beam radiation therapy (EBRT), brachytherapy, androgen deprivation therapy (ADT), watchful waiting, and active surveillance, used alone or in combination (1–4).

Conventional EBRT for LPC consists of a total dose of 76–81 Gy (1.8–2.0 Gy dose/fx) delivered over approximately 8–9 weeks (4).

Considering logistical problems and their impact on quality of life, this long treatment can be difficult to deal with for many patients. Taking this into account, thanks to technological evolution and favorable prostate cancer radiobiology, the overall treatment time has been increasingly reduced thanks to the use of hypofractionation (dose/fx > 2.0 Gy) (5–9).

Several publications suggest a radiobiological rationale for hypofractionated radiotherapy in prostate cancer treatment (10–12).

The low α/β ratio of prostate cancer (13, 14), lower than in near-normal tissues (bladder and rectum), suggests a high sensitivity to dose per fraction of cancer cells and is therefore advantageous in terms of efficacy and tolerance of hypofractionation treatment. Trials demonstrated that moderate hypofractionation (fraction sizes from 2.5 Gy to 3.5 Gy) is effective without greater toxicity than conventional EBRT (5–9, 15). Also, ablative or stereotactic hypofractionation (daily fractions of 6–10 Gy), exploiting the postulated radiobiological advantage, has been increasingly used in the treatment of LPC (16–28). The adoption of stereotactic body radiotherapy (SBRT) in the treatment of LPC has been possible thanks to several technological improvements, allowing the delivery of a carefully high dose or fraction thanks to precise localization of the target and decreasing the toxicity to organs at risk (OARs). There are several dedicated SBRT linear accelerators (LINACs), but the CyberKnife^®^ (CK) System (Accuray Incorporated, Sunnyvale, CA, USA) has been used in most SBRT experiences in LPC. The CyberKnife^®^, a linear accelerator mounted on a robotic device, has the possibility of tracking the prostate during the treatment in real-time, thanks to three to four intraprostatic gold fiducials, and then adapting the delivery of the treatment on the basis of the geometric information received by these fiducials. This feature, with a targeting error of less than 1 mm, allows for a reduction in the target volumes and better limits the dose to surrounding organs at risk (29). The data of CK SBRT in LPC at a total dose of 35–36.25 Gy in five fractions demonstrate excellent biochemical control rates with early and late toxicity profiles similar to those of conventional EBRT (21–26). In this study, we reported our initial experience with SBRT using CyberKnife in the treatment of localized prostate cancer (the CyPro Trial). The goal of this study is to evaluate the effectiveness of CK SBRT in terms of biochemical disease-free survival, early and late rectal and urinary toxicities, sexual toxicity, and quality of life.

## 2 Materials and methods

### 2.1 Patient selection

CyPro (CyberKnife Prostate cancer) is a trial comprising a retrospective part, presented in this work, and a prospective part that is still ongoing. In both, patients undergoing re-irradiation are also evaluated. In this article, retrospective data of patients treated for the first time are presented.

The patients with biopsy-proven localized prostate adenocarcinoma (transrectal core biopsy with at least 10 cores) underwent CyberKnife^®^ SBRT as the primary treatment.

They were enrolled according to D’Amico risk stratification: low risk (T2a or lower stage, prostate-specific antigen (PSA) ≤10 ng/ml and Gleason score ≤6) and intermediate risk (T2b-2c, PSA ≤ 20 ng/ml and Gleason score 7 (3 + 4)). Patients with Gleason scores of 7 (4 + 3) were excluded. Maximum prostate volume eligible was ≤90 cc. Our protocol did not include ADT, but a percentage of patients received it for up to 3 months as prescribed by the urologist. No patients continued androgen deprivation during or after radiotherapy. For TNM stage evaluation, multiparametric prostate magnetic resonance image (MRI) (if contraindicated, CT pelvic was performed), CT abdomen and chest, and bone scintigraphy were performed.

### 2.2 Trial approval

We conducted this study within the CyPro Trial (CyberKnife Prostate cancer) prot. 46/19, approved by the Ethics Committee on 15.01.2020 (D. n. 105 of 12.02.2020). We present data about the retrospective CyPro Trial. Before the SBRT, the patients were informed and given an informed choice on the implantation of four gold fiducial markers and stereotactic radiotherapy with the CyberKnife^®^ System.

### 2.3 Planning and delivery

Four gold fiducial markers were implanted transperineally into the prostate with rectal ultrasound control in a triangular-like configuration. The fiducials were tracked during each fraction, including translations and rotations, and beam aim automatically corrected when motion was detected.

At 7 days or 10 days after fiducial placement, patients underwent a non-contrast simul-CT scan (1-mm cuts) in a supine position with a personalized immobilization system (Vac-Lok). Target volume delineation was performed using a simul-CT scan with prostate MRI fusion. Gross target volume (GTV) was defined as prostate for low risk and as prostate plus proximal 2-cm seminal vesicles for intermediate risk. Clinical target volume (CTV) was equal to the GTV. Planning target volume (PTV) was defined as CTV with a 3-mm expansion posteriorly and 5 mm in all directions. The rectum, bladder, penile bulb, femoral heads, bowel, testicles, and neurovascular bundle were contoured as OARs.

The treatment planning was performed using the Multiplan^®^ inverse treatment planning system (Accuray Inc., Sunnyvale, CA, USA). The prescription dose was 35–36.25 Gy to the PTV, delivered in five fractions of 7–7.25 Gy (EQD2 85–90 Gy with α/β ratio of 1.5), normalized to the 80% isodose line, and covered at least 95% of the PTV. The constraints for OARs are presented in **Table 1**.

**Table 1:** Organ at risk, maximum dose, and dose limit at organ at risk.

OAR	Dmax	Dose limit
***Bladder***		V37Gy < 10 cc V37.5Gy < 5 cc V50% < 40% V100% < 10%
***Rectum***	<38 Gy	V36Gy < 1 cc V25Gy < 20 cc V50% < 50% V80% < 20% V90% < 10% V100% < 5%
***Penile bulb***		V29Gy < 50% V30Gy < 3 cc
***Femoral head***		V14.5Gy <5% (bilateral)
***Testes***		D20% < 2 Gy
***Neurovascular bundle***		V49Gy < 10%
***Bowel***		V29.5Gy < 10% V30Gy < 1 cc

OAR, organ at risk.

Patients were irradiated on alternate days using the CyberKnife^®^ System, with a 6-MV photon beam linear accelerator installed on a six-degrees-of-freedom robotic arm connected to a six-degrees-of-freedom robotic couch. The system, with two orthogonal kilovoltage X-ray images, tracks during the treatment (in real-time) the position of intraprostatic fiducials and, based on the geometric information received by these, adapts the delivery of the beams and corrects the position of the patient.

### 2.4 Follow-up, toxicity, and quality of life evaluation

Rectal and urinary acute/late toxicities were scored on the Radiation Therapy Oncology Group (RTOG) toxicity scale (28) during the treatment, at 3 months post-treatment intervals during the first 2 years, and at 6-month intervals thereafter.

Acute toxicities were defined as any adverse event occurring within 3 months of the treatment, and late toxicities were defined as any adverse events after 3 months from the treatment.

The quality of life (QoL) was assessed using the European Organisation for Research and Treatment of Cancer (EORTC) QLQ C30, Global Health Status (GHS) and PR25, International Erectile Function Index-5 (IIEF5), and International Prostate Symptom Score (IPSS) questionnaires administered at baseline, every 3 months post-treatment during the first year, and at 18 months and 24 months thereafter.

The PSA level was obtained at baseline and prospectively at 3 months post-treatment intervals during the first year and at 6-month intervals thereafter. Disease-free survival (DFS) was defined as the time from treatment of the tumor to loss of local control or primitive disease distant progression. Biochemical disease-free survival (bDFS) was defined using the Phoenix method (nadir + 2 ng/ml) (29). SBRT-related outcomes were analyzed in the patients with a follow-up of 3 months at least.

### 2.5 Statistical analysis

Patient characteristics were reported as medians and ranges for continuous variables and percentages for categorical variables.

To analyze actuarial outcomes, we used the Kaplan–Meier method; differences among subgroups were evaluated by log-rank tests. Statistical analysis was carried out by SPSS statistical software (IBM Corp. Released 2011. IBM SPSS Statistics for Windows, Version 20.0. Armonk, NY: IBM Corp).

## 3 Results

### 3.1 Patient and treatment characteristics

From February 2013 to December 2019, CK SBRT was performed in 122 patients with LPC, mean age 70 years (range 46–88): 53 low risk (LR) and 69 intermediate risk (IR). Twenty-six patients also received ADT. Seventy-one patients were treated with 35 Gy in five fractions (7 Gy/fx), and 51 patients were treated with 36.25 Gy in five fractions (7.25 Gy/fx). Typically, 271 (95 to 550) non-coplanar beams were used in each treatment session, and the median time of delivery was 40 min (range 38–50 min).

Patient and treatment characteristics are in **Table 2**.

**Table 2:** Patient and treatment characteristics.

	Total patients (122)	Patients treated with dose 35 Gy/5 fx (71)	Patients treated with dose 36.25 Gy/5 fx (51)
Age at first RT visit (years)			
Mean ± SD	70.3 ± 6.6	70.4 ± 6.8	70.2 ± 6.3
Median	71.5	72	71
(range)	(46–88)	(46–84)	(56–88)
Age at first RT visit, n (%)			
≤65	26 (0.21)	13 (0.18)	13 (0.25)
66–70	29 (0.24)	17 (0.24)	12 (0.24)
>70	67 (0.55)	41 (0.58)	26 (0.51)
PSA level at diagnosis (ng/ml)			
Mean ± SD	7.7 ± 3.5	8.1 ± 3.7	7.3 ± 3.2
Median	6.9	7	6.8
(range)	(1.91–16.60)	(2.49–16.50)	(1.91–16.60)
PSA level at diagnosis, n (%)			
≤10	98 (0.80)	54 (0.76)	44 (0.86)
>10 and <20	24 (0.20)	17 (0.24)	7 (0.14)
PSA level pre-treatment (ng/ml)			
Mean ± SD	6.7 ± 4.0	6.5 ± 4.3	7.1 ± 3.6
Median	6.5	6.4	6.9
(range)	(0.07–17.28)	(0.07–17.28)	(0.19–15.22)
PSA level pre-treatment, n (%)			
≤10	99 (0.81)	59 (0.83)	40 (0.78)
>10 and <20	23 (0.19)	12 (0.17)	11 (0.22)
Risk group, n (%)			
Low	53 (0.43)	31 (0.44)	22 (0.43)
Intermediate	69 (0.57)	40 (0.56)	29 (0.57)
Hormone treatment, n (%)			
Yes	26 (0.21)	24 (0.34)	2 (0.04)
No	96 (0.79)	47 (0.67)	49 (0.96)
TURP before SBRT, n (%)			
Yes	8 (0.07)	6 (0.08)	2 (0.04)
No	114 (0.93)	65 (0.92)	49 (0.96)
Site RT, n (%)			
Prostate	53 (0.43)	31 (0.44)	22 (0.43)
Prostate+SV	69 (0.57)	40 (0.56)	9 (0.57)

RT, radiotherapy; TURP, transurethral resection prostate; SBRT, stereotactic body radiotherapy.

### 3.2 Follow-up, biochemical control, and quality of life

The median follow-up was 4 years (range, 3–60 months).

**Figure 1:**
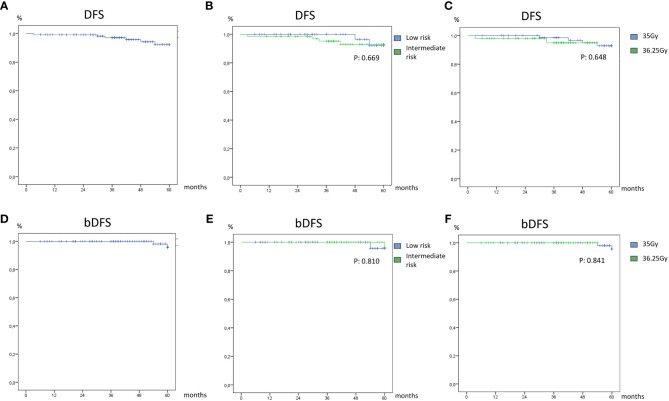
Disease-free survival **(A)** and biochemical disease-free survival **(D)** of overall patients’ low **(B)** and intermediate risk **(E)** and dose treatment of 35 Gy **(C)** and 36.2 Gy **(F)**.

The 1-, 2-, and 5-year DFS rates were 92.9%, 92.9%, and 92.3%, respectively, while the 1-, 2-, and 5-year bDFS rates were 100%, 100%, and 95.7%, respectively. Regarding risk groups or doses, no statistically significant difference was found in terms of DFS or bDFS (**Figure 1**).

In particular, two patients showed biochemical relapse at 42 months and 54 months, respectively; another two patients showed a recurrence in the lymph node and prostatic gland at 30 months and 48 months, respectively, through the use of radiological exams; one patient showed biochemical and instrumental recurrence 3 months after the treatment.

In terms of overall survival (OS), the 1-, 2-, and 5-year OS rates were 99.2%, 98.2%, and 86.1%, respectively. Also, in this case, no statistically significant difference was found for risk groups (*p* = 0.338) or doses (*p* = 0.338).

Twelve patients died from causes unrelated to prostatic cancer: seven from heart disease (acute myocardial infarction), four from a second tumor (one lung, one gastric, one renal, and one hepatocellular carcinoma), and one from gastrointestinal disease.

**Figure 2:**
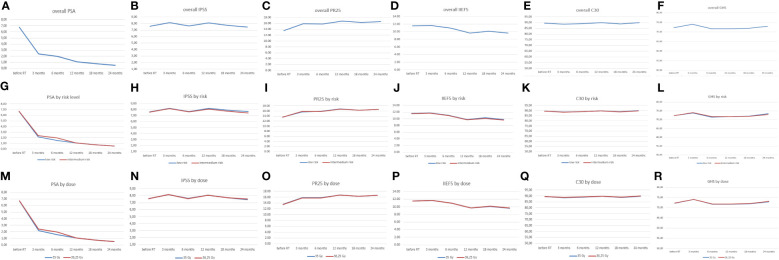
Median value of PSA, IPSS, PR25, IIEF5, C30, and GHS in overall patients (line blu **A-F**) and patients divided by class of risk (line blue low-risk and red intermediate-risk **G-L**) and by dose levels (blue line 35Gy and red line 36.25Gy **M-R**). PSA, prostate specific antigen; IPSS, International Prostate Symptom Score; IIEF5, International Erectile Function Index-5; GHS, Global Health Status.

The mean and medium values of PSA, IPSS, PR25, IIEF5, CR30, and GHS at the different time points (pre-RT and 3 months, 6 months, 12 months, 18 months, and 24 months from the RT) are reported in **Figure 2** and is reported in more details in **Table S1** in the **Supplementary Material**.

The PSA mean value decreased statistically significantly at each time point (data not shown), except for the decrease between 3 and six months, which was at the significance limit (*p* = 0.054). In terms of risk and doses, there was no statistically significant difference, except for the values of PSA at 3 months divided by dose levels (**Figure 2M**), although in this case the result could be influenced by the higher percentage (34%) of patients in the 35-Gy/5 fz group who received ADT before radiotherapy, compared to the percentage in the 36.25-Gy/fz group (4%).

Regarding the IPSS, the mean value changed in a statistically significant way at each time point (*p* < 0.001); no statistically significant difference was found at each point for different risk levels, while a statistically significant difference was found at 12 months and 24 months between different dose levels (**Figure 2N**).

For PR25, the mean value changed in a statistically significant way at each time point (*p* < 0.001); no statistically significant difference was found at each point for different risk levels, while a statistically significant difference was found at 18 months between different dose levels (**Figure 2O**).

The IIEF5 score changed in a statistically significant way at each time point (*p* < 0.001); no difference was found in terms of risk levels, but there was a difference in terms of doses between the mean values at 6 months, 12 months, 18 months, and 24 months from the RT (**Figure 2P**). Similarly, the C30 and GHS showed a statistically significant change at each time point (*p* < 0.001). No statistically significant difference was found at each time point for risk levels for both the parameters; however, a statistically significant difference was found in GHS at 12 months, 18 months, and 24 months for dose levels (**Figure 2R**).

### 3.3 Toxicity

**Figure 3:**
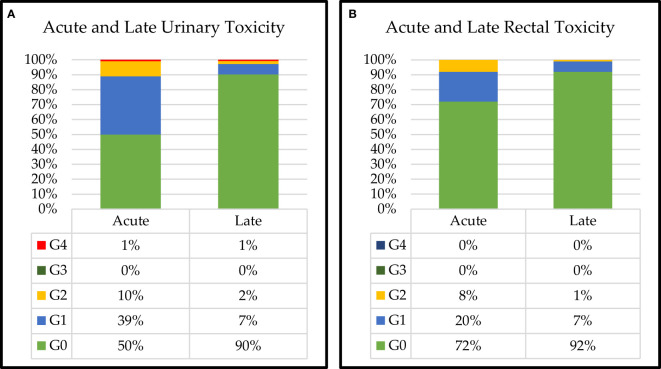
Acute and late **(A)** urinary and **(B)** rectal toxicities for all patients.

Acute and late urinary and rectal toxicity profiles were collected for all patients (**Figures 3A, B**). Grade 2 urinary toxicity was acute in 10% and late in 2% of patients. No Grade 3 acute and late urinary toxicity was detected (30, 31).

We found 1% of acute and late urinary toxicity in Grade 4.

Grade 2 rectal toxicity resulted in acute in 8% and late in 1% of patients. There was no Grade 3–4 acute and late rectal toxicity.

**Figure 4:**
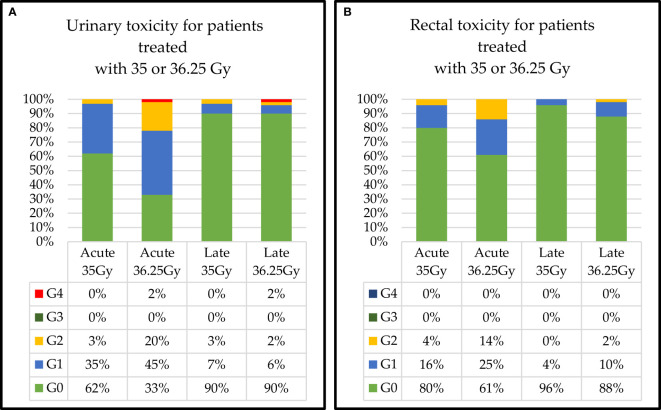
Dose-related acute and late **(A)** urinary and **(B)** rectal toxicities.

Dose-related acute and late urinary toxicities are shown in **Figure 4A**. Grade 2 acute toxicity appeared higher in the high-dose group (20% in the 36.25-Gy group *vs.* 3% in the 35-Gy group) and was not statistically significant (data not shown). No Grade 3 acute and late toxicities were detected in the two groups, while Grade 4 acute and late toxicity (obstructive symptoms) of 2% was found in the 36.25-Gy group. Dose-related acute and late rectal toxicities are shown in **Figure 4B**. Grade 2 toxicity appeared higher in the 36.25-Gy group than in the 35-Gy group (acute 14% *vs.* 4% and late 2% *vs.* 0%). No Grade 3 or higher acute or late rectal toxicity was detected in both groups.

**Figure 5:**
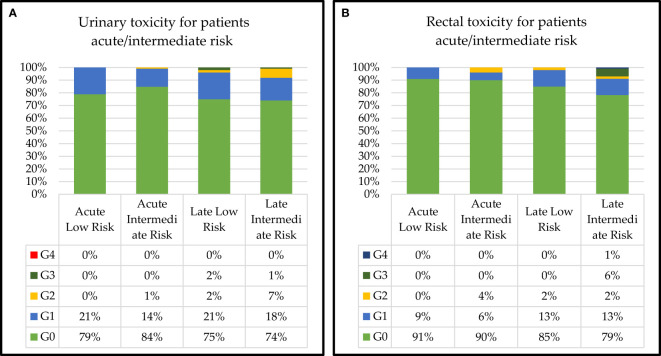
Class of risk-related acute and late **(A)** urinary and **(B)** rectal toxicities.

The class of risk-related acute and late urinary toxicities is shown in **Figure 5A**. Grade 2 acute and late toxicity appeared higher in the intermediate-risk group (1% *vs.* 0% and 7% *vs.* 2%, respectively) but was not statistically significant (data not shown). Grade 3 acute toxicities were detected in two groups: 2% in low risk and 1% in intermediate risk. No Grade 4 acute and late toxicity was found in both groups. Dose-related acute and late rectal toxicities are shown in **Figure 5B**. Grade 2 acute toxicity was 2% higher in the intermediate group (4% *vs.* 0%). Grade 2 late toxicity was 2% in both groups. Regarding late rectal toxicity, we recorded 6% of Grade 3 and 1% of Grade 4 (rectal bleeding requiring intensive care) in the intermediate group.

## 4 Discussion

Over the past 14 years, many studies of SBRT for early prostate cancer, in which a total of 35–50 Gy was administered in four or five fractions, have been published (19, 21–24, 27, 28).With variable follow-up duration, these studies, mainly involving low- and intermediate-risk patients treated with the CyberKnife System, showed excellent biochemical control with low toxicity profiles (32, 33). These favorable results are probably related to the low alpha/beta ratio of prostate cancer, which, if considered 1.5 Gy, would demonstrate that 35–36.25 Gy in five fractions is equivalent to a dose of 90–95 Gy at 1.8 Gy per fraction or 200–212-Gy biologically effective dose (BED) (14).

Our study confirms that SBRT of 35–36.25 Gy in five fractions with the CyberKnife System yields excellent control with low toxicity in low–intermediate-risk prostate cancer patients.

We recorded in the lower-dose group an earlier achievement of the nadir and a more significant reduction in the PSA value at the nadir, but the result could be influenced by the higher percentage of patients in the 35-Gy/5 fz group who received ADT (not included in our protocol) prescribed by urologists a few months before. We found no dose-related differences in biochemical control or overall survival. In the same setting, we might find similar results. For example, Katz recorded a 6-year reported outcome and toxicity data in an initial evaluation. The bDFS was 97% for low-risk, 90.7% for intermediate-risk, and 74.1% for high-risk patients, with no difference in bDFS as a function of dose for low-risk patients (21). In a 7-year analysis, Katz, expanding the number of previous studies, reported that in 477 prostate cancer patients treated with CyberKnife SBRT at a total dose of 35 Gy/5 fx (154 points) and 36.25 Gy/5 fx (323 points), the bDFS rate was 95.6% and 89.6% for the low- and intermediate-risk groups, respectively (*p* < 0.012). In an updated 10-year analysis of 230 low-risk patients showing a bDFS of 93.7% and a median PSA of 0.1 ng/ml, the same author found that the different doses of 35 Gy and 36.25 Gy had no impact on biochemical control (25).

King et al. performed a pooled analysis on 1,100 clinically localized prostate cancer patients who underwent CK SBRT at a mean dose of 36.25 Gy (35–40 Gy/4–5 fx), and the 5-year bDFS was 95%, 84%, and 81% for low-, intermediate-, and high-risk patients, respectively (*p* < 0.001) (24). Other authors evaluating the dose–response relationships for PSA decay and biochemical recurrence noted that an increased dose was associated with greater prostate ablation and PSA decay. Meier et al. (28) in a multi-institutional series and Tree et al. (34) found excellent 5-year biochemical relapse-free survival (bRFS) rates of 95% or greater for low-risk diseases.

In our experience, there were no significant differences in toxicity or quality of life between the two groups. Grade 2 urinary toxicity was acute in 10% and late in 2% of patients. Grade 2 rectal toxicity resulted in acute symptoms in 8% and late symptoms in 1% of patients. There were no acute or late urinary or rectal toxicities in Grades 3–4. Grade 2 acute toxicity appeared higher in the high-dose group (20% in the 36.25-Gy group *vs.* 3% in the 35-Gy group) but was not statistically significant. Literature confirms limited toxicities. Katz reported that in patients treated with 35 Gy, a Grade 2 late urinary toxicity of 4% and a Grade 2 late rectal toxicity of 2% were present; in patients treated with 36.25 Gy, a Grade 2 late urinary toxicity of 9% and a Grade 2 late rectal toxicity of 5% were present (21).

In an analysis with a longer follow-up period, Katz noted Grade 2 late urinary toxicity in 9% and Grade 3 in 3%. Late Grade 2–3 urinary toxicity appeared higher in the high-dose group (4% at 35 Gy *vs.* 15% at 36.25 Gy), with a statistically significant difference (*p* = 0.07), while there was no clear difference in late rectal toxicity rates between the two doses. Bolzicco, in his experience, recorded that late Grade 1, 2, and 3 urinary toxicities occurred in 4%, 3%, and 1% of the patients, respectively, while late Grade 1 rectal toxicity occurred in two patients and Grade 2 toxicity in one patient; no Grade 3 or 4 late rectal toxicities were observed (26).

Arscott et al., in a 2-year evaluation, reported acute and late urinary retention in Grade 2 at 39.5% and 41.4%, respectively. A mean baseline IPSS-obstructive score of 3.6 significantly increased to 5.0 at 1 month (*p* < 0.0001) and returned to baseline in 92.6% of cases within a median time of 3 months (35).

Other authors, such as Loblaw, recorded a 1% rate of late severe genitourinary (GU) (temporary catheterization in a patient with a 300-cm^3^ bladder diverticulum) and gastrointestinal (GI) toxicities (anal fistula in a patient with background diverticulitis) (36, 37).

In conclusion, the reported toxicities are also low, with late Grade 3 GU and GI toxicities usually less than 2%. The main exception to this was seen in a dose escalation study of up to 50 Gy in five fractions, which reported 7% and 6% rates of Common Terminology Criteria for Adverse Events (CTCAE) v. 3.0 late Grade 3 GI and GU toxicities, respectively, including Grade 4 cystitis requiring ureteroileal diversion, Grade 4 rectal bleeding, and six patients who required a colostomy (38).

Katz et al. and McBride et al. revealed that they were significantly more likely to develop late rectal toxicity if the rectal wall received V50Gy >3 cm^3^, >35% of the rectal wall circumference received 39 Gy, and >50% of the rectal wall circumference received 24 Gy.

Finally, in our experience with erectile dysfunction, we found no differences based on dose, but we did find them in relation to the age of the patients.

Regarding QoL, Katz found the mean EPIC QoL scores for the bowel and urinary domains decreased initially, then returned to baseline at 1 year, and remained so for up to 8 years. The EPIC sexual scores decreased by 40%. There was no significant difference in bowel, sexual, or urinary EPIC scores between 35 Gy and 36.25 Gy at any time point (25). In a subset of the patients, King et al. (n = 864) evaluated quality of life using the EPIC tool. The decline in quality of sex life was predominantly observed within the first 9 months, a pattern unaffected by the use of androgen deprivation therapy or patient age (39).

Dixit evaluated the health-related QoL outcomes among 45 prostate cancer patients following SBRT with CyberKnife. The mean GHS score improved from 81.3 at baseline to 82.4 at 6 weeks and was 75.6 at 6 months (*p* > 0.05, not significant). EORTC PR25 and C30 scores did not reveal any significant change from the baseline (40).

Quality of life data also appear consistent across the literature, with initial deterioration over the first few months in the urinary and bowel domains, followed by subsequent recovery to baseline over the next 6 months to 12 months (41, 42).

## 5 Conclusions

In conclusion, our data, in line with the literature data, seem to confirm that hypofractionated SBRT for prostate cancer appears to be an excellent therapeutic option for patients with low- and intermediate-risk diseases. It has very low toxicities and high patient compliance, which may make it superior to conventional fractionation and other radiotherapy techniques. Further confirmation of these results is awaited through the prospective phase of this trial, which is still ongoing, and through a longer-term follow-up.

## Data availability statement

Original datasets are available in a publicly accessible repository: Zotero. The original contributions presented in the study are publicly available. The data will be available as raw data to URL: Stereotactic Body Radiotherapy with Cyberknife® System for Low-and Intermediate-Risk Prostate Cancer. Clinical Outcomes and Toxicities of CyPro Trial —Zenodo (DOI 10.5281/zenodo.8187900, accessed on 26 Jul 2023).

## Ethics statement

We conducted this study within the CyPro Trial (CyberKnife Prostate cancer) prot. 46/19, approved by the Ethics Committee on 15.01.2020 (D. n. 105 of 12.02.2020). The studies were conducted in accordance with the local legislation and institutional requirements. Written informed consent for participation was not required from the participants or the participants’ legal guardians/next of kin in accordance with the national legislation and institutional requirements. Written informed consent was obtained from the individual(s) for the publication of any potentially identifiable images or data included in this article.

## Author contributions

RDF: Conceptualization, Methodology, Writing – original draft, Writing – review & editing. VB: Conceptualization, Data curation, Writing – original draft, Writing – review & editing. ES: Conceptualization, Data curation, Supervision, Writing – review & editing. DP: Writing – original draft, Conceptualization, Data curation, Formal Analysis, Methodology. MS: Writing – review & editing, Investigation, Methodology, Software, Validation. GA: Writing – original draft, Conceptualization, Data curation, Methodology, Validation. GQ: Investigation, Methodology, Supervision, Validation, Writing – original draft. SPe: Writing – review & editing, Conceptualization, Methodology, Supervision, Validation. SR: Conceptualization, Supervision, Validation, Writing – original draft. SPi: Supervision, Writing – original draft. AC: Writing – original draft, Conceptualization, Data curation, Formal Analysis, Methodology. PG: Writing – review & editing, Data curation, Formal Analysis, Investigation, Methodology, Software. VD’A: Methodology, Software, Validation, Writing – review & editing. CA: Methodology, Writing – review & editing, Conceptualization, Formal Analysis. FB: Conceptualization, Investigation, Supervision, Validation, Writing – review & editing. SM: Conceptualization, Data curation, Methodology, Writing – original draft. AR: Conceptualization, Supervision, Writing – review & editing. AT: Conceptualization, Supervision, Validation, Writing – original draft. PM: Conceptualization, Supervision, Validation, Writing – original draft.
