# Supplementary material for: Stereotactic body radiotherapy with CyberKnife® System for low- and intermediate-risk prostate cancer: clinical outcomes and toxicities of CyPro Trial
Source: Front Oncol. 2023 Nov 7;13:1270498. doi: 10.3389/fonc.2023.1270498 (PMC10660677; doi:10.3389/fonc.2023.1270498)
Supplement: Supplementary file 1 [file Table_1.docx]

| **PSA** | | | | | | | |  | **IIEF5** | | | | | | | |
| --- | --- | --- | --- | --- | --- | --- | --- | --- | --- | --- | --- | --- | --- | --- | --- | --- |
|  | | before RT | 3 months | 6 months | 12 months | 18 months | 24 months |  |  | | before RT | 3 months | 6 months | 12 months | 18 months | 24 months |
| **overall** | mean | 6,73 | 2,40 | 1,96 | 1,06 | 0,76 | 0,52 |  | **overall** | mean | 13,00 | 13,00 | 12,00 | 10,00 | 10,00 | 9,50 |
|  | median | 6,48 | 1,84 | 1,14 | 0,64 | 0,50 | 0,31 |  |  | median | 11,54 | 11,63 | 10,96 | 9,68 | 10,10 | 9,64 |
| **low risk** | mean | 6,67 | 2,17 | 1,53 | 1,06 | 0,73 | 0,51 |  | **low risk** | mean | 13,00 | 13,00 | 12,00 | 10,00 | 10,50 | 10,00 |
|  | median | 6,38 | 1,80 | 1,13 | 0,64 | 0,45 | 0,30 |  |  | median | 11,58 | 11,71 | 11,02 | 9,78 | 10,30 | 9,77 |
| **intermediate risk** | mean | 6,73 | 2,40 | 1,96 | 1,06 | 0,76 | 0,52 |  | **Intermediate risk** | mean | 13,00 | 13,00 | 12,00 | 10,00 | 10,00 | 9,50 |
|  | median | 6,48 | 1,84 | 1,14 | 0,64 | 0,50 | 0,31 |  |  | median | 11,54 | 11,63 | 10,96 | 9,68 | 10,10 | 9,64 |
| **35 Gy** | mean | 6,68 | 2,20 | 1,55 | 1,07 | 0,74 | 0,52 |  | **35 Gy** | mean | 13,00 | 13,00 | 12,00 | 10,00 | 10,00 | 9,50 |
|  | median | 6,40 | 1,80 | 1,14 | 0,66 | 0,49 | 0,31 |  |  | median | 11,54 | 11,63 | 10,96 | 9,68 | 10,10 | 9,64 |
| **36,25 Gy** | mean | 6,73 | 2,40 | 1,96 | 1,06 | 0,76 | 0,52 |  | **36,25 Gy** | mean | 13,00 | 13,00 | 12,00 | 10,00 | 10,00 | 10,00 |
|  | median | 6,40 | 1,80 | 1,14 | 0,64 | 0,50 | 0,31 |  |  | median | 11,54 | 11,63 | 10,96 | 9,68 | 10,13 | 9,66 |
| **IPSS** | | | | | | | |  | **C30** | | | | | | | |
| **overall** | mean | 6,00 | 6,00 | 6,00 | 6,00 | 6,00 | 6,00 |  | **overall** | mean | 93,00 | 93,00 | 93,00 | 94,00 | 92,50 | 93,00 |
|  | median | 7,57 | 8,14 | 7,61 | 8,09 | 7,70 | 7,46 |  |  | median | 89,42 | 88,59 | 89,13 | 89,82 | 88,88 | 89,88 |
| **low risk** | mean | 6,00 | 6,00 | 6,00 | 6,00 | 6,00 | 6,00 |  | **low risk** | mean | 93,00 | 93,00 | 93,00 | 94,00 | 93,00 | 93,00 |
|  | median | 7,63 | 8,22 | 7,65 | 8,23 | 7,87 | 7,66 |  |  | median | 89,41 | 88,73 | 89,11 | 89,82 | 88,95 | 90,12 |
| **Intermediate risk** | mean | 6,00 | 6,00 | 6,00 | 6,00 | 6,00 | 6,00 |  | **Intermediate risk** | mean | 93,00 | 93,00 | 93,00 | 94,00 | 92,50 | 93,00 |
|  | median | 7,57 | 8,14 | 7,61 | 8,09 | 7,70 | 7,46 |  |  | median | 89,42 | 88,59 | 89,13 | 89,82 | 88,88 | 89,88 |
| **35 Gy** | mean | 6,00 | 6,00 | 6,00 | 6,00 | 6,00 | 6,00 |  | **35 Gy** | mean | 93,00 | 93,00 | 93,00 | 94,00 | 92,50 | 93,00 |
|  | median | 7,57 | 8,14 | 7,61 | 8,09 | 7,70 | 7,46 |  |  | median | 89,42 | 88,59 | 89,13 | 89,82 | 88,88 | 89,88 |
| **36,25 Gy** | mean | 6,00 | 6,00 | 6,00 | 6,00 | 6,00 | 6,00 |  | **36,25 Gy** | mean | 93,00 | 93,00 | 93,00 | 94,00 | 93,00 | 93,00 |
|  | median | 7,57 | 8,15 | 7,59 | 8,07 | 7,70 | 7,51 |  |  | median | 89,48 | 88,65 | 89,19 | 89,90 | 88,95 | 89,98 |
| **PR25** | | | | | | | |  | **GHS** | | | | | | | |
| **overall** | mean | 13,00 | 14,00 | 14,00 | 15,00 | 16,50 | 15,00 |  | **overall** | mean | 75,00 | 83,00 | 75,00 | 75,00 | 75,00 | 75,00 |
|  | median | 13,54 | 15,85 | 15,77 | 16,77 | 16,33 | 16,60 |  |  | median | 72,21 | 74,02 | 71,70 | 71,72 | 71,93 | 72,93 |
| **low risk** | mean | 13,00 | 14,00 | 14,00 | 15,00 | 15,00 | 15,00 |  | **low risk** | mean | 75,00 | 83,00 | 75,00 | 75,00 | 75,00 | 79,00 |
|  | median | 13,63 | 15,56 | 15,93 | 16,82 | 16,33 | 16,66 |  |  | median | 72,31 | 73,74 | 71,39 | 71,71 | 72,00 | 73,39 |
| **Intermediate risk** | mean | 13,00 | 14,00 | 14,00 | 15,00 | 16,50 | 15,00 |  | **Intermediate risk** | mean | 75,00 | 83,00 | 75,00 | 75,00 | 75,00 | 75,00 |
|  | median | 13,54 | 15,85 | 15,77 | 16,77 | 16,33 | 16,60 |  |  | median | 72,21 | 74,02 | 71,70 | 71,72 | 71,93 | 72,93 |
| **35 Gy** | mean | 13,00 | 14,00 | 14,00 | 15,00 | 16,50 | 15,00 |  | **35 Gy** | mean | 75,00 | 83,00 | 75,00 | 75,00 | 75,00 | 75,00 |
|  | median | 13,54 | 15,85 | 15,77 | 16,77 | 16,33 | 16,60 |  |  | median | 72,21 | 74,02 | 71,70 | 71,72 | 71,93 | 72,93 |
| **36,25 Gy** | mean | 13,00 | 14,00 | 14,00 | 15,00 | 16,00 | 15,00 |  | **36,25 Gy** | mean | 75,00 | 83,00 | 75,00 | 75,00 | 75,00 | 79,00 |
|  | median | 13,51 | 15,71 | 15,68 | 16,68 | 16,30 | 16,60 |  |  | median | 72,26 | 74,08 | 71,75 | 71,76 | 72,03 | 73,16 |

**Supplementary Material**

**Table 1s.** Mean and Medium values of PSA, IPSS, PR25, IIEF5, CR30, and GHS at pre-RT and 3 months, 6 months, 12 months, 18 months and 24 months from the RT
